# Interpretation and content validity of the items of the numeric rating version short-WORC to evaluate outcomes in management of rotator cuff pathology: a cognitive interview approach

**DOI:** 10.1186/s12955-020-01339-7

**Published:** 2020-03-30

**Authors:** Rochelle Furtado, Joy C. MacDermid, Dianne M. Bryant, Kenneth J. Faber, George S. Athwal

**Affiliations:** 1 Physiotherapy, Health and Rehabilitation Science, Western University, London, ON Canada; 2 Collaborative Program in Musculoskeletal Health Research, Bone and Joint Institute, Western University, London, ON Canada; 3 Roth McFarlane Hand and Upper Limb Centre, St. Joseph’s Hospital, London, ON Canada

**Keywords:** Patient reported outcomes, Rotator cuff disorders, Content validity, Quality of life

## Abstract

**Background:**

The shortened version of the Western Ontario Rotator Cuff Index (Short-WORC) is a patient reported outcome measure that evaluates quality of life (QoL) of patients with rotator cuff pathology. However, formal content validation of the full or Short-WORC has not been reported. This study aims to understand how 1) people interpret and calibrate responses to items on the Short-WORC and 2) compensatory strategies that might enhance function and thereby affect responses.

**Methods:**

This study uses cognitive interviewing, a qualitative methodology that focuses on the interpretation of questionnaire items. Patients with rotator cuff disorders (*n* = 10), clinicians (*n* = 6) and measurement researchers (n = 10) were interviewed using a talk aloud structured interview that evaluated each of the 7 items of the Short-WORC. All interviews were recorded and transcribed verbatim by one researcher (R.F). Analysis was done through an open coding scheme using a previously established framework.

**Results:**

Overall, the items on the Short-WORC were well received by participants. Through the interviews, the 6 themes of: Comprehension, Inadequate response definition, Reference Point, Relevance, Perspective Modifiers and Calibration Across Items emerged. The items of working above the shoulder (90%), compensating with the unaffected arm (88%) and lifting heavy objects (92%) were the most relevant to participants. Participants calibrated their scores on the items of sleeping and styling (19%) the most. Perspective modifiers of gender, influenced the calibrations of items of styling your hair (30%) and dressing or undressing (19%). Compensatory strategies of task-re allocation and using assistive devices/resources were frequently mentioned by participants. Overall, participants had minor comprehension issues, but found the 7- items of the Short-WORC to be relevant to QoL.

**Conclusions:**

Therefore, the findings demonstrate that the Short-WORC is not cognitively complex, but varies with patient perspectives. Overall, the Short-WORC provides evidence of demonstrating strong content validity when used for rotator cuff disorder patients.

## Introduction

Rotator cuff disorders (RCDs) include a spectrum of pathologies that can lead to shoulder pain, impairment and activity limitation [1]. While the spectrum of disorders vary, rotator cuff tears are a common problem in the current patient population. Rotator cuff tears are commonly associated with exposure to repetitive movements or strain [1, 2]. The prevalence of tears increases with age, affecting more than 60% of patients who are over the age of 60 [3] and results in a reduced quality of life (QoL) [1].

The construct of QoL is critical for defining optimal treatments, as the goal of surgery and rehabilitation is to improve the QoL in patients [3, 4]. The previous version of the Western Ontario Rotator Cuff Index (WORC) developed by Kirkley et al., is one of the most validated disease-specific questionnaire to measure QoL in RCDs [5]. The WORC focuses on 5 domains; 1) pain and physical symptoms, 2) sports and recreation, 3) work, 4) lifestyle, and 5) emotions [5]. While it has been translated and validated in a variety of different languages [1, 2], the WORC created challenges of patient response burden (time spent to answer questionnaire) and complexity identified through patient interviews and statistical methods [1, 2] A shortened version of the Western Ontario Rotator Cuff Index (Short-WORC) was created to address these concerns [6, 7]. The Short-WORC by Razmjou et al. contains seven items from the domains of work and lifestyle, focusing on the activity limitations that arise from RCDs [1, 4, 6, 8]. Previously, participants completed a visual analogue scale (VAS) to score their response [6], however, in the present study we have modified the responsiveness scale to a 0–10-point numeric scale. According to previously literature, the use of a numeric scale reduces patient response burden and increases patient satisfaction [9]. As previously mentioned in prior work supporting the Short-WORC [1, 8], the construct of QoL may not be fully retained in this abbreviated questionnaire. In fact, it seems the Short-WORC is assessing the construct of activity limitation and function. Nevertheless, in our preliminary studies and those of others, the Short-WORC has demonstrated measurement properties that are similar to the original WORC [1, 8]. While the Short-WORC demonstrates equally strong psychometric performance when items are extracted from the full WORC [1, 8], it has yet to be validated as an independent outcome measure in a clinical population [7].

A fundamental aspect of validation is understanding the content validity of a questionnaire [10]. Content validity refers to the extent to which a measure represents all facets of a given construct [11]. When evaluating a shortened PRO, it is advised that while some properties can be obtained from the original study, the property of comprehensiveness should be evaluated from a new study of the shortened PRO [12]. Therefore, it is important that researchers re-evaluate the content validity of a shortened PRO, to verify that it measures the intended construct of the original. According to the Food and Drug Administration’s (FDA) guidelines [13], content validity can be assessed through conducting interviews that seek to evaluate 1) the clarity of the instructions, 2) the content of each item and 3) that the intended meaning of each item is easily interpreted by participants. In this process, the recall and response scales of the PRO are also evaluated [10, 11]. Therefore, this study primarily aims to evaluate content validity by exploring how people interpret and calibrate responses to items on the Short-WORC, in a population of rotator cuff disorders. A secondary aim of this study is to understand how compensatory strategies may influence the way participants interpret and determine responses to the items.

## Methods

### Study design

This study used a descriptive qualitative approach based on the principles of cognitive interviewing to explore participants’ interpretations of specific words, constructs (variables that cannot be measured directly but are informed through other variables that are measurable) and phrases on the Short-WORC. This enables an understanding of how participants calibrate options when responding to the measure [14, 15]. Cognitive interviewing uses semi-structured interviews, a talk out loud approach, and probes to understand how patients interpret and respond to items on a self-report questionnaire [14]. These items measure quality of life on the questionnaire. This allows a combination of concurrent (while answering the question) or retrospective (immediately after answering the item) answering, which gathers optimal data quality [14]. Participants were provided with a version of the Short-WORC, that had a numerical scale from 0 to 10.

### Setting and sample

Interviews were conducted in a small private room at the Hand and Upper Limb Clinical (HULC) Research Laboratory, London, Canada. Patient and healthcare provider participants were recruited from St. Joseph’s Health Care London and researchers were recruited from Western University (London, Canada). Participants who met the inclusion criteria were invited to participate in the study; greater than 18 years of age, can speak and read English and did not have another mental or physical aliment that could contraindicate the shoulder injury or not allow them to be able to participate in the interview process. This questionnaire was introduced into standard routine care of core outcome measures for shoulders at HULC. Patients only answered questions about the Short-WORC and no other PRO.

Through purposeful sampling, we aimed to include perspectives of healthcare providers and recipients [16]. Therefore, patients(*n* = 10), healthcare providers (*n* = 6), and measurement researchers (n = 10) were recruited. Patients who had received some treatment (i.e. surgery, physiotherapy) for their rotator cuff disorder at HULC (*n* = 6) and patients who had been diagnosed at HULC but not received treatment for their shoulder (*n* = 4) were recruited to achieve a diversity of participant experiences. Patients had a variety of rotator cuff disorders. Diagnoses ranged from under a year (*n* = 4) for patients who have not received treatment, and from 1 year to 5 years of diagnosis (*n* = 6) for patients who had received treatment. Healthcare providers were a mix of both sexes and occupations included: nurses, surgeons, physiotherapists and occupational therapists practicing in London, Ontario. Measurement researchers included trainees of both sexes in the faculty of Health and Rehabilitation Sciences at Western University. Trainees have had exposure to questionnaire design and evaluating psychometric properties. Participation of both men and women of varying age groups allowed for a diversity of experiences. Recruitment for interviews stopped when saturation of the responses was achieved [14]. The study protocol was reviewed and approved by Lawson Healthcare and Western Research Ethics Board (WREB).

### Data collection

Participants provided written informed consent prior to the interview. Interviews were conducted in English by one researcher (RF) and lasted between 40 and 60 min. All interviews were recorded on an encrypted tape recorder, and then transcribed verbatim.

The interview structure was informed by previously published work [14, 17] and multiple discussions with the research team. Interviews focused on participants’ interpretation of each individual item on the Short-WORC. Through the think out loud approach, participants were encouraged to express all their thoughts when responding to each item. Probes such as, “Can you define this word?” or “Can you provide me with an example?”, were asked to further explore the rationale of participants’ specific responses to each item. Participants described how they determined (calibrated) their responses.

### Analysis

Descriptive statistics (age, sex, occupational status and diagnosis) were collected and are presented in Table 1. The original audio recordings were analyzed by the research team. Analysis of the recordings were done through a descriptive thematic analysis, consisting of open coding [14]. This allowed the scripts to be characterized by fragments, in order for relevant themes to be extracted, categorized and classified first. Next, themes were identified from the responses to each item. Findings were summarized with quotes and percentages as appropriate. A previously established coding system [18] was used to classify issues that affect interpretation. The categories include: Comprehension/clarity (C), Perspective modifiers (PM), Reference point(RP), Calibration across items(CAI), Inadequate response definition (IR) and Relevance (R).

**Table 1:** Demographic results of study population (*n* = 26)

Groups	Patients who have received treatment (***n*** = 6)	Patients yet to be exposed to treatment (***n*** = 4)	Clinicians (***n*** = 6)	Measurement Researchers (***n*** = 10)
Mean age (years)	76	44	34	31
Female (%)	67	50	50	50
Male (%)	33	50	50	50
Professional status (%)				
Student	0	25	33	100
Employed	33	50	67	0
Unemployed	0	0	0	0
Sick-leave	0	0	0	0
Retired	67	25	0	0
Affected shoulder (%)				
Left	33	25	N/A	N/A
Right	50	25	N/A	N/A
Bilateral	17	50	N/A	N/A
Diagnosis (%)				
Shoulder pain	67	75	N/A	N/A
Rotator cuff tear	67	25	N/A	N/A
TSA	67	0	N/A	N/A
OA	67	0	N/A	N/A

## Results

Data analysis resulted in the categorization of 6 themes that describe the issues that participants had when interpreting items. This is further described and illustrated by participants’ quotes, as demonstrated in Table 2.

**Table 2:** Common issues that arose with the Short-WORC (*n* = 26)

Item	Comprehension	Perspective Modifiers	Reference Point	Calibration Across Items	Inadequate Response definition	Relevance
1. Sleeping	Understood well					Overall, found to be relevant (73%) Few participants felt it was only relevant if the injured arm was slept on.
2. Styling your hair	Understod well	Gender issues arose (30%). E.x. men who were bald found this inapplicable		Some participants calibrated this answer with the item of sleeping (19%)		Overall, relevant to 79% of participants
3. Dressing or undressing	Understood well	Some gender issues arose, i.e. men who did not rate this item as important (19%)		Some participants calibrated this answer with the item of sleeping and styling hair (19%)		Overall, relevant to 70% of participants
4. Daily activities about the house or yard	Some comprehension issues with defining daily activities and yard work					Overall was relevant for 80% of participants 12% found work in the yard irrelevant to their Qol
5. Working above the shoulder	Understood well					Overall, 90% of participants found it was relevant to their QoL
6. Compensate with unaffected arm	Understood well					88% of participants found this relevant to their QoL. 12% found if the injury was on the non-dominant hand it was not relevant.
7. Lifting heavy objects at or below shoulder level	Understood well					92% of participants found this to be relevant to QoL

Overall findings demonstrate that individuals interpreted items based on their personal situation, personality traits, biology, work roles and/or environmental factors [17], which was represented through the theme of perspective modifiers [19]. Certain items such as, styling your hair or dressing, were more influenced by gender. While the genders found this item relevant to QoL, males assigned less importance to this item when compared to items such as, working above shoulder level. Additionally, perspective modifiers also influenced the relevance of doing work outside the house for participants who specified living in an apartment or having designated help prior to the injury. Therefore, item relevancy to the construct of QoL is influenced by a person’s biological, environmental or social context.

### Short-WORC items

#### Instructions on short- WORC

The Short-WORC focuses on two different domains which have unique sets of instructions. When participants (*n* = 26) were asked to read the instructions out loud, some comprehension (C) issues arose. Specifically, there was misinterpretation around some of the important words in the instructions. For example, when asked to define the word “past week”, some participants confused this with “average week”.

*“Okay so here I have to answer and think about my lifestyle and what I do on an average day in the week, and how much my shoulder has been kinda affecting or altering those activities.” – Measurement Researcher #1, female*

Some participants also struggled with the interpretation of the word “lifestyle”. When asked to define, the majority of participants associated lifestyle with only activities of daily living.

*“Lifestyle is my activities during the day. Would the term activities of daily living be better suited instead of lifestyle, is this what you are asking?” - Clinician #4, female*

##### How much difficulty do you have sleeping because of your shoulder?

Overall, this item was well understood by most participants and did not demonstrate comprehension issues. When asked to define the term “sleeping”, frequent words such as: “at rest, relaxed at night and lying down” arose. The majority of participants (all groups - 73%) considered this item to be relevant to their lifestyle, however, some participants in the patient group suggested that it was only relevant if they slept on the injured shoulder.

*“I always sleep on my left side so my right shoulder (injured) is fine at night.”- Patient #8, female*

Additionally, some participants in the patient group (19%) described compensator strategies that allowed them to sleep better at night. Participants in the patient groups further discussed themes of intense shoulder pain, which translated to modifications of their sleeping position to comfortably rest.

“*Since the surgery was on my left shoulder, my left shoulder was out, and so I mostly slept on my back*.” – patient #4, male.

##### How much difficulty have you experienced with styling your hair because of your shoulder?

Comprehension of this item was generally clear to participants in all three groups. When asked to define the term “styling your hair”, phrases of “grooming, blow drying, combing, brushing and using styling products” arose amongst all three groups. Findings demonstrate this item to be relevant to QoL with the majority of participants (all groups- 79%), however, the theme of perspective modifiers heavily influenced the interpretation.

Relevance was a gendered issue with this item. Men in the patient group more often found this item to be irrelevant to their QoL as they had short hair or were bald (30%).

*“I’m bald, I don’t need to style my hair!” – Patient #5, male.*

Furthermore, some participants in the group of patients who had not received treatment (19%), used the item of sleeping, as a reference point to calibrate their response to the item of styling your hair. Additionally, female participants in the patient group and measurement researcher group identified that styling their hair was critical to their QoL, and so needed to compensate with the uninjured arm, seek assistance or allot more time in their day for styling.

“*If I were to injure my shoulder, I would still style my hair …I would get someone (roommate) to just help me out if I needed a specific style.”- Measurement Researcher #1, female.*

##### How much difficulty do you have dressing or undressing?

In general, definitions of “dressing or undressing” were interpreted as “putting on clothes, removing clothes, and getting ready”, indicating no comprehension issues with the participant group of patients. Findings further indicated that the patient participants (70%) strongly endorsed the relevance of this item to their QoL. Additionally, some participants in the patient group and measurement researcher group (30%) calibrated their response to this item, based on their scores for the items of sleeping and styling their hair.

“*I would say my answer would be the same as styling my hair…if I chose 5 or 6 in question 2, then I would choose the same answer for question 3.” – Measurement Researcher #2, male*

Furthermore, participants in the patient group identified the importance of completing this task and the need to compensate to complete it. Strategies for compensation included: requiring assistance from a device or family member, increasing the allotted time for changing of clothes, or changing the types of clothing worn in order to decrease shoulder movement.

*“I can’t reach my back to put on my bra…that’s why my husband helps me out.” – Patient #6, female.*

##### How much difficulty do you experience in daily activities about the house or yard?

Findings indicated some comprehension issues with this item, as participants would interchange the words “lifestyle” and “daily activities” often. When asked to define “daily activities”, terms such as: “chores, work, school, and living style” frequently arose amongst all participant groups.

“*Yeah my daily activities are defined by my work and hobbies. My life is my job, family and other activities I do.” – Patient #5, female.*

In contrast, definitions of “about the house or the yard” resulted in phrases of: “chores, eating, cleaning, cooking, gardening and yard /outdoors work”. Only one female participant in the clinician group initially misinterpreted the meaning of about the house or yard, and defined it as occupational labour that involves working outdoors.

*“This means work outside of the house like employment that you get paid for or yard work. This is both inside or outside the house and external jobs…that’s what I think”- Clinician #6, Female*

Overall, all participants in all groups (80%) identified this item to be relevant to their overall quality of life, but some (12%) were concerned with the phrasing of “work in the yard”. Due to patient participants’ living conditions, some did not require the need to do yard work, i.e. living in an apartment or having designated help prior to the injury.

*“No, I do not do any yard work, my husband always does that.” – Patient #8, female.*

Additionally, some compensatory strategies were mentioned from patients such as: seeking assistance from someone else to do their daily activities or modifying the time period or frequency of activities they participated in.

##### How much difficulty do you experience with working above the shoulder?

Overall, this item received positive feedback from all participants in all groups (90%), as many identified this item to be a critical component of recovery. Some female participants in the patient group (10%) identified that they did not need to do much overhead reaching and therefore, found this item less important to their quality of life.

“*I have an office job, I don’t need to raise my arms much.” – Patient #8, female.*

Definitions of “working above the shoulder” included phrases such as: “overhead reaching, lifting above my head and raising my arms”, indicating comprehension was generally good for this item. Participants in the patient group frequently mentioned compensating strategies in order to continue to work above shoulder level, such as: modifying the placement of items for easier access or seeking assistance when needing to reach above shoulder level.

*“I try to use my left hand a lot more to help out and then I keep things within reach. The shelves are much lower in my house and if something is too high for me I use a step ladder.” – Patient #10, female.*

##### How much do you use your unaffected arm to compensate for the injured arm?

Definitions of “compensate” led to phrases such as: “using my not injured shoulder and using my healthy shoulder more”, indicating no comprehension issues amongst participants. Furthermore, this item was identified as a critical component for QoL by participants in all groups (88%).

*“I use my left hand a lot, which is much harder since I am very right-hand dominant.” – patient #6, female*.

In contrast, some female participants in the patient group (12%) indicated that compensating was less relevant, as their injury was on their non-dominant arm.

*“I am right handed; my injury was on my left shoulder…do I compensate? Not frequently”.*

*– Patient #3, female*.

##### How much difficulty do you experience lifting heavy objects at or below shoulder level?

When asked to define “heavy objects”, participants all groups stated words such as: “weight, large and using force”, indicating the item was well understood. Overall, this item resulted in a mix of responses depending on what stage of recovery the participant was in. Participants in the patient group who were further along their recovery scored this item lower, while participants who were in the early stages of the injury scored it higher. Nevertheless, the majority of participants (92%) identified this item to be important to QoL. While evident that participants in the patient group understood this item, some (12%) participate in a variety of tasks below shoulder level and therefore, were unsure which tasks to calibrate their score to.

*“I do some yard work and cleaning that can be difficult to bend and pick up things from time to time...I think something in the middle?”- Patient, #3, female*

Additionally, some participants in the patient group discussed compensatory strategies such as re-allocating the task to someone else in order to feel less discomfort.

*“Now with the snow coming, I will have to shovel myself since the weather is bad and I will have to find help.”- Patient #7, female.*

## Discussion

Overall, the content validity of the Short-WORC was supported within this population, as most respondents found the items on the Short-WORC to be clear and relevant to their functioning. However, the item of styling your hair was not relevant to a minority of the study sample and had a gender-bias being less relevant to men. Furthermore, it was evident that many patients had developed compensatory strategies, as this was mediated in the difficulty reported. Overall, the items received positive feedback, there was no struggle with the recall period, and most of the items were correctly interpreted.

According to the **CO**nsensus-based **S**tandards for the selection of health **M**easurement **IN**struments (COSMIN), content validity is one of the most important criterion for evaluating a PROs, and should be assessed by both patients and professionals [20, 21]. In 2018 [22], COSMIN defined the standards for adequate content validity to be a measure that is comprehensive, comprehensible and relevant [12, 22, 23]. Comprehension of the items is also an important component of content validation according to the International Society for Pharmacoeconomics and Outcomes Research (ISPOR) [24]. Findings indicate that the comprehension level of the Short-WORC is adequate for the intended population. Furthermore, is it recommended to researchers to use appropriate language that does not diverge from the intended meaning [16, 17, 25].

Another component of cognitive interviewing is the evaluation of participants’ recall period. The recall period, assesses participants’ responses based on the strategies they use when responding to an item [14]. The results confirm the lack of difficulty participants had in the recall phase when responding to the items, and there was no further indication of any unclear reference boundaries that could have impaired their responses.

In addition, another facet of construct validation is item relevancy. Items must be relevant to the intended population and construct being assessed [14]. While results demonstrated a high percentage of relevancy to participants, none of the items were relevant to every individual participant. As anticipated, individuals are unique and have different opinions of what they calibrate as relevant to their QoL or recovery. While researchers try to anticipate this issue in the development of PROs, variables such as: gender, age, lifestyle, or social status will always hinder the relevancy of an item, as they are not generalizable [16]. In the original iteration of the WORC, Kirkley et al. [5] used factor analysis and semi-structured interviews to rank the relevancy of items when measuring QoL. Therefore, researchers should use a variety of analytical methods during item selection to enhance the relevancy of items to the construct and the participants.

We conclude that the Short-WORC has shown to support content validation, based on the evidence presented in this study, and within the intended population. Findings demonstrate that all items were relevant to the majority of participants when evaluating their QoL, and the issues were relatively minor. A minority of participants found the item of *styling your hair* to be not relevant to their lifestyle, the majority of participants did. Changing existing measures is a major undertaking since it creates confusion and makes it less possible to compare data across time. Therefore, major issues should be present to warrant these changes. According to COSMIN guidelines [12, 21, 22], there is no reason to remove the item, but certain words could be replaced to improve clarity. Overall, it is evident that the comprehension levels of the Short-WORC were easy enough for all participants, and no major comprehension issues were identified that would result in the removal of items. Finally, it is evident that the recall period was accurately evaluated and participants found no difficulty in that process. Therefore, it is evident that the Short-WORC demonstrates aspects of content validation in the intended population, and qualitatively supports aspects of our prior work [1, 6, 8].

A secondary purpose of this study was to explore the compensatory strategies that influence the participants calibrate their responses. Findings demonstrated strategies of modifying activity levels, lowering personal expectations or re-allocating tasks in order to avoid stress to the shoulder. Understanding the compensatory strategies for RCDs, provide further insight into why participants with similar impairments might report different levels of functional ability [17, 26]. Further, understanding compensatory mechanisms can provide insight into potential for other injuries [24]. For example, overcompensation with the uninjured arm, may increase the risk for an injury in the uninjured arm. Therefore, it is important for researchers to understand the compensatory strategies that might enhance function and affect responses during item development [24, 27].

Overall, limitations in this study included the use of a population that was predominately middle-class Caucasian. However, the demographic region from where this study was conducted is predominately of Caucasian descent [19, 28]. Therefore, futures studies should gather information from other ethnic groups in order to compare and contrast the QoL. Additionally, future directions could explore validating the Short-WORC quantitatively within the intended population of interest, through the use of the content validity index [29] to account for this limitation. Furthermore, while the WORC was designed to measure quality of life, the Short-WORC focuses on activity limitation. Therefore, this study cannot be taken as supporting that the WORC and Short-WORC demonstrate aspects of concurrent content validity within the current patient population.

## Conclusion

In conclusion, the evidence in this study demonstrates that there is no need for change to the items of the Short-WORC, as they tend to be understood by patients with rotator cuff disorders. The Short-WORC reflects the principles of comprehension, relevance and recall, supporting aspects of content validation in the intended population. Overall, the items on the Short-WORC are able to capture aspects of activity limitation, and should be used with a numerical scale from 0 to 10. Future studies should assess other psychometric properties such as reliability, validity and responsiveness prospectively.

## Data Availability

All data generated or analyzed during this study are included in this published article.

## Abbreviations

C: Comprehension
CAI: Calibration across items
COSMIN: **CO**nsensus-based **S**tandards for the selection of health **M**easurement **IN**struments
FDA: Food and Drug Administration
HULC: Hand and Upper Limb Clinical Research Laboratory
IR: Inadequate response definition
ISPOR: International Society for Pharmacoeconomics and Outcomes Research
PM: Perspective modifier
PRO: Patient reported outcome
QoL: Quality of Life
R: Relevance
RCD: Rotator cuff disorder
RP: Reference point
Short-WORC: Shortened version of the Western Ontario Rotator Cuff Index
VAS: Visual analogue scale
